# Exploring the interplay of vitamin D, salivary antimicrobial peptides, and cytokines in oral immunity and disease prevention: an insight for implications in oral health policy

**DOI:** 10.3389/froh.2025.1694969

**Published:** 2026-01-21

**Authors:** Ashwini Tumkur Shivakumar, Sumana Mahadevaiah Neelambike, Supreeta R. Shettar, G. K. Megha, Sowmya Halasabalu Kalgeri, Varsha D. Shiragannavar, Nirmala G. Sannappa Gowda, Dhakshaini MR, Keshava Prasad, Prasanna K. Santhekadur

**Affiliations:** 1 Department of Conservative Dentistry and Endodontics, JSS Dental College and Hospital, JSSAHER, Mysore, India; 2 Department of Microbiology, JSS Medical College and Hospital, JSSAHER, Mysuru, India; 3 Department of Biochemistry, JSS Medical College and Hospital, JSSAHER, Mysuru, India; 4 Department of Biochemistry, MVJ Medical College and Research Hospital, Hoskote, India; 5 Department of Prosthodontics and Crown and Bridge, JSS Dental College and Hospital, JSSAHER, Mysore, India; 6 Department of Conservative Dentistry and Endodontics, RV Dental College and Hospital, Bengaluru, India

**Keywords:** β-defensin 2, cathelicidin, metabolic syndrome, salivary AMPs, vitamin D

## Abstract

**Introduction:**

Oral diseases such as caries and periodontitis are complex, multifactorial diseases and remain the most prevalent worldwide. Both diseases are associated with vitamin D deficiency (VDD) and its pathophysiological process. The prevalence of metabolic syndrome (MetS) is rising in developing countries, and studies have shown that oral diseases are more prevalent among the metabolic group. This study aims to assess and correlate the relationship between serum vitamin D levels, salivary antimicrobial peptides (AMPs), and their role in oral health.

**Materials and methods:**

A total of 80 individuals aged 35–75 years were recruited, including 40 patients in the metabolic syndrome group, following the National Cholesterol Education Program Adult Treatment Panel III (NCEP ATP III) criteria, and 40 in the non-metabolic group. Serum vitamin D levels and salivary AMPs were estimated using enzyme-linked immunosorbent assay (ELISA). Oral health status was assessed using the Decayed, Missing, Filled Teeth (DMFT) index and by recording the presence or absence of periodontal pockets. The data obtained were subjected to statistical analysis to determine correlations between systemic status, biochemical markers, and oral health parameters.

**Results:**

The Mann–Whitney *U* test was applied to compare continuous variables between the metabolic and non-metabolic groups, as well as between subgroups. The chi-squared test was applied to assess associations between categorical variables. Patients with metabolic syndrome exhibited reduced serum vitamin D levels, although differences were not statistically significant. Salivary β-defensin and cathelicidin levels were relatively higher in the metabolic group, but without significant intergroup variation. A higher DMFT score and greater prevalence of periodontal pockets were recorded among metabolic syndrome patients compared with non-metabolic individuals.

**Conclusions:**

Within the study's limitations, a potential link between metabolic syndrome and compromised oral health was observed, as demonstrated by increased caries incidence and periodontal involvement. While variations in serum vitamin D and salivary AMPs did not achieve statistical significance, the findings suggest an altered host defense mechanism in metabolic syndrome patients. Larger, longitudinal studies are required to further elucidate these associations and emphasize the importance of routine oral screening in individuals with metabolic disorders, and the implementation of oral health polices is mandatory to maintain good oral health and well-being among both metabolic and non-metabolic populations.

## Introduction

The awareness of the activity of vitamin D, which primarily acts as a steroid hormone, has increased in the past few decades. It is essential to have an adequate synthesis of vitamin D for the normal function of the other systems in the body (1). Vitamin D is a multifunctional hormone that is synthesized when the skin is exposed to sun rays; a tiny amount of it is supplied from external sources such as foods and supplements. Vitamin D is a broad name that comprises vitamin D2 and D3 (2). It acts as an autocrine and paracrine agent, influencing and controlling cell differentiation, cell maturation, and the innate immune system (3). The cellular action of vitamin D is mediated by the presence of vitamin D receptors (VDR) (4). It organizes the physiological functions by managing the calcium and phosphate metabolism, promotes growth, and induces the necessary remodeling of the bones and teeth (5).

Oral diseases such as caries and periodontal diseases are complex and multifactorial and remain the most prevalent diseases worldwide (6). Both diseases are associated with vitamin D deficiency (VDD) and its pathophysiological process (7). The oral environment is unique; the oral mucosa serves as a protective barrier against microbes from both the inner and outer environments. The oral epithelium, saliva, and neutrophils contribute to oral health by producing antimicrobial peptides (AMPs), which help protect the oral cavity from pathogens (8). Strong oral immune responses help prevent dental caries, periodontal disease, and systemic infections originating from the oral cavity (9). Antimicrobial peptides are naturally found in the components of saliva, oral epithelium, and neutrophils. Literature has revealed that antimicrobial peptides help in balancing oral health (10).

Metabolic syndrome (MetS) is characterized by the combination of one or more systemic conditions (11). The metabolic syndrome is a clustering of several conditions, such as hyperglycemia/insulin resistance, obesity, dyslipidemia, high triglyceride levels, and low high-density cholesterol levels (12). Metabolic syndrome is associated with increased risk of periodontal disease, tooth loss, and impaired oral health due to chronic inflammation, insulin resistance, and altered saliva composition. Poor oral health can, in turn, exacerbate systemic inflammation, creating a bidirectional relationship (13). The prevalence of metabolic syndrome has risen in developing countries. Studies have linked poor oral hygiene and vitamin D deficiency among metabolic syndrome patients (14).

There is limited data among the Indian population exploring the association between vitamin D, metabolic syndrome, and oral health. Few studies have investigated these aspects independently, with a minimal focus on salivary AMPs and their role in oral health. Hence, this study aims to evaluate the association between vitamin D and antimicrobial peptides, such as cathelicidin (CAMP) and β-defensin 2, their relationship in maintaining oral health, and their inverse relationship with tumor necrosis factor alpha (TNF-α) among metabolic and non-metabolic patients. The findings will also provide insights to inform the development of oral health policies aimed at improving overall health among metabolic and non-metabolic populations.

## Materials and methods

A cross-sectional analytical study was conducted among patients visiting the tertiary healthcare center in Mysuru, after obtaining the Institutional Ethics Committee approval, with the IEC no. 13/2021.

The study's sample size was calculated using Cohen's *d* to estimate the standardized effect size for two-group comparisons. A significance level of *α* = 0.05 and a statistical power of 80% were applied to ensure reliable detection of differences. Considering prior research and clinical significance, a medium-to-large effect size (*d* ≈ 0.6–0.7) was chosen to capture meaningful variations in oral health outcomes between groups. A minimum of 40 participants per group is required, and the final calculated sample size was 80.

Patients visiting the dental outpatient department were evaluated and included in the study. Informed consent was obtained from the patients, who were willing to be a part of the study. Non-metabolic and metabolic patients classified according to the NCEP ATP III criteria (15) (Table 1), aged between 35 and 75 years, were considered for the study. Subjects on vitamin D supplements for the last 6 months, smokers, and tobacco chewers were excluded. A total of 80 patients were included in the study, with 40 patients in each of the metabolic and non-metabolic groups.

**Table 1:** Criteria to diagnose MetS according to WHO and NCEP ATP III (15)

NCEP ATP III criteria (metabolic syndrome)		
	Component	Cutoff criteria
1	Waist circumference	Men: >102 cm (40 inches) Women: >88 cm (35 inches)
2	Triglycerides (TG)	≥150 mg/dL OR on treatment for high TG
3	HDL cholesterol	Men: <40 mg/dL Women: <50 mg/dL
4	Blood pressure	≥130/85 mmHg OR on antihypertensive medication
5	Fasting blood glucose	≥100 mg/dL OR on treatment for elevated glucose (e.g., anti-diabetic medication)

**Table 2:** Comparison of serum vitamin D levels among the metabolic and non-metabolic groups.

Vitamin D levels	Metabolic group	Non-metabolic group	*p*-value
Range	11.44–96.59	36.62–96.28	0.577
Mean ± SD	52.24 ± 23.07	52.47 ± 12.04	
Deficient	11 (27.5%)	2 (5%)	
Sufficient	23 (57.5%)	37 (92.5%)	
Higher than normal	6 (15%)	1 (2.5%)	

Test applied: Mann–Whitney *U* test; no statistically significant difference observed (*p* > 0.05).

### Collection of the blood sample

A 5 mL blood sample was collected. The samples were centrifuged for 20 min at approximately 1,000 × *g* and stored at −80°C. The level of Vitamin D3 was analyzed using the ELISA method according to the instructions in the manual (16).

### Collection of the saliva sample

Patients were seated comfortably in the dental chair and were instructed to rinse their mouths with saline. Five milliliters of saliva were then collected by the spitting method into a sterile container. All the samples were stored at −20°C till the experiment was carried out. The samples were brought to room temperature and centrifuged for 20 min at 1,000 × *g* before the assays. The supernatant was obtained and assayed immediately. The samples were tested for cathelicidin antimicrobial peptide (CAMP), β-defensin 2, and tumor necrosis factor alpha (TNF-α).

### Oral health status

Oral health was evaluated by recording the Decayed, Missing, Filled Teeth (DMFT) index along with the presence and absence of pockets. The William probe was used to record the depth, where the periodontal pocket depth, >3 mm, was considered as the presence of a pocket.

## Results

Data were statistically analyzed using IBM SPSS Statistics for Windows, Version 20.0. Descriptive statistics were used, with continuous variables presented as mean ± standard deviation (SD) and median with interquartile range (IQR) and categorical variables expressed as frequencies and percentages. The normality of continuous data was assessed using the Shapiro–Wilk test. As most variables did not follow a normal distribution, non-parametric tests were selected. The Mann–Whitney *U* test was used for comparison of continuous variables between metabolic and non-metabolic groups, as well as between gender and periodontal subgroups. The chi-squared test was used to assess associations between categorical variables.

Spearman's rank correlation coefficient was applied to evaluate relationships between serum vitamin D, salivary antimicrobial peptides, TNF-α, and oral health indices (DMFT components, periodontal pockets). All tests were two-tailed, and a *p*-value of <0.05 was considered statistically significant.

Serum vitamin D levels did not differ significantly between the metabolic and non-metabolic groups. Although the mean values were comparable, a relatively higher proportion of deficiency was observed in the metabolic group (27.5%) compared with that in the non-metabolic group (5%). Conversely, the majority of participants in the non-metabolic group exhibited sufficient vitamin D levels (92.5%). Despite this apparent variation in distribution, there was no significant difference (Table 1).

The level of salivary cathelicidin exhibited no significant difference between the metabolic and non-metabolic groups. The mean ± SD was 35.73 ± 12.75 ng/mL in the metabolic group and 35.52 ± 11.42 ng/mL in the non-metabolic group. The median values were also comparable, with 39.91 ng/mL (IQR: 29.37–44.86) in the metabolic group and 38.23 ng/mL (IQR: 31.78–42.05) in the non-metabolic group. The distribution of values across both groups was broadly similar (Table 3).

**Table 3:** Comparison of salivary cathelicidin (CAMP) levels among the metabolic and non-metabolic groups.

Cathelcidin (camp), ng/mL	Range	Mean ± SD	Median (IQR)	*p*-value
Metabolic group	0.8–50.62	35.73 ± 12.75	39.91 (29.37–44.86)	0.658
Non-metabolic group	7.89–50.66	35.52 ± 11.42	38.23 (31.78–42.05)	

Test applied: Mann–Whitney *U* test; no statistically significant difference observed (*p* > 0.05).

Salivary β-defensin levels were marginally higher in the metabolic group, with a mean of 0.72 ± 0.61 ng/mL and a median of 0.55 ng/mL (IQR: 0.16–1.23), compared with those in the non-metabolic group, showing a mean of 0.51 ± 0.48 ng/mL and a median of 0.33 ng/mL (IQR: 0.12–0.80). However, there was no significant difference, and the range of values overlapped considerably between the two groups (Table 4).

**Table 4:** Comparison of salivary β-defensin levels among the metabolic and non-metabolic groups.

β-Defensin, ng/mL	Range	Mean ± SD	Median (IQR)	*p*-value
Metabolic group	−0.07 to 2	0.72 ± 0.61	0.55 (0.16–1.23)	0.179
Non-metabolic group	0.03–1.9	0.51 ± 0.48	0.33 (0.12–0.8)	

Test applied: Mann–Whitney *U* test; no statistically significant difference observed (*p* > 0.05).

The mean salivary TNF-α level was 0.46 ± 0.35 ng/mL in the metabolic group and 0.62 ± 0.42 ng/mL in the non-metabolic group. The median values followed a similar trend, with 0.38 ng/mL (IQR: 0.15–0.64) and 0.64 ng/mL (IQR: 0.23–0.84) in the respective groups. Although TNF-α levels appeared slightly higher in the non-metabolic group, the difference was not statistically significant (Table 5).

**Table 5:** Comparison of salivary TNF-α levels among the metabolic and non-metabolic groups.

TNF-α, ng/mL	Range	Mean ± SD	Median (IQR)	*p*-value
Metabolic group	0.03–1.44	0.46 ± 0.35	0.38 (0.15–0.64)	0.121
Non-metabolic group	0.02–1.5	0.62 ± 0.42	0.64 (0.23–0.84)	

Test applied: Mann–Whitney *U* test; no statistically significant difference observed (*p* > 0.05).

The analysis of DMFT components revealed notable differences between the metabolic and non-metabolic groups. The median number of total affected teeth (DMFT score) was higher in the metabolic group (5; IQR: 3–6) compared with the non-metabolic group (3; IQR: 1–4), and this difference was statistically significant (*p* = 0.004), suggesting a greater cumulative burden of dental caries experience among individuals with metabolic syndrome (Table 6).

**Table 6:** Comparison of DMFT components between the metabolic and non-metabolic groups.

DMFT	Metabolic group		Non-metabolic group		*p*-value
	Range	Median (IQR)	Range	Median (IQR)	
Decayed teeth	0–8	1.5 (0.25–3.75)	0–6	1 (0–2)	0.163
Missing teeth (due to caries)	0–11	2 (0–3.75)	0–12	0 (0–1)	<0.0001^a^
Filled teeth (due to caries)	0–5	0 (0–0)	0–4	0 (0–1)	0.203
Total number of permanent teeth affected	0–13	5 (3–6)	0–14	3 (1–4)	0.004^a^

Test applied: Mann–Whitney *U* test

^a^ Indicates statistical significance (*p* < 0.05).

When analyzed individually, the number of missing teeth due to caries showed a significant difference, with the metabolic group presenting a higher median value of 2 (IQR: 0–3.75) compared with 0 (IQR: 0–1) in the non-metabolic group (*p* < 0.0001). This may reflect a higher rate of tooth loss among those with metabolic disturbances, possibly due to chronic inflammation, delayed healing, or other systemic influences.

Although the median number of decayed and filled teeth did not differ significantly between the groups, slightly higher values were observed in the metabolic group. These trends, while not statistically significant, may still suggest subtle oral health differences associated with metabolic status.

Periodontal pocket in the posterior region, presented with a statistically significant difference between the groups, with 71.4% of affected individuals belonging to the metabolic group compared with 28.6% in the non-metabolic group (*p* = 0.020). In the anterior region, although a higher proportion of pocket presence was observed in the metabolic group (75%), it was not statistically significant (*p* = 0.057). These findings may indicate a trend toward greater periodontal involvement in individuals with metabolic syndrome, particularly in the posterior region (Table 7).

**Table 7:** Distribution of periodontal pocket presence among the metabolic and non-metabolic groups.

Pocket		Metabolic group	Non-metabolic group	*p*-value
Anterior	Yes	9 (75%)	3 (25%)	0.057
	No	31 (45.6%)	37 (54.4%)	
Posterior	Yes	15 (71.4%)	6 (28.6%)	0.020^a^
	No	25 (42.4%)	34 (57.6%)	

Test applied: chi-squared test

^a^ Indicates statistical significance (*p* < 0.05).

## Discussion

**Figure 1:**
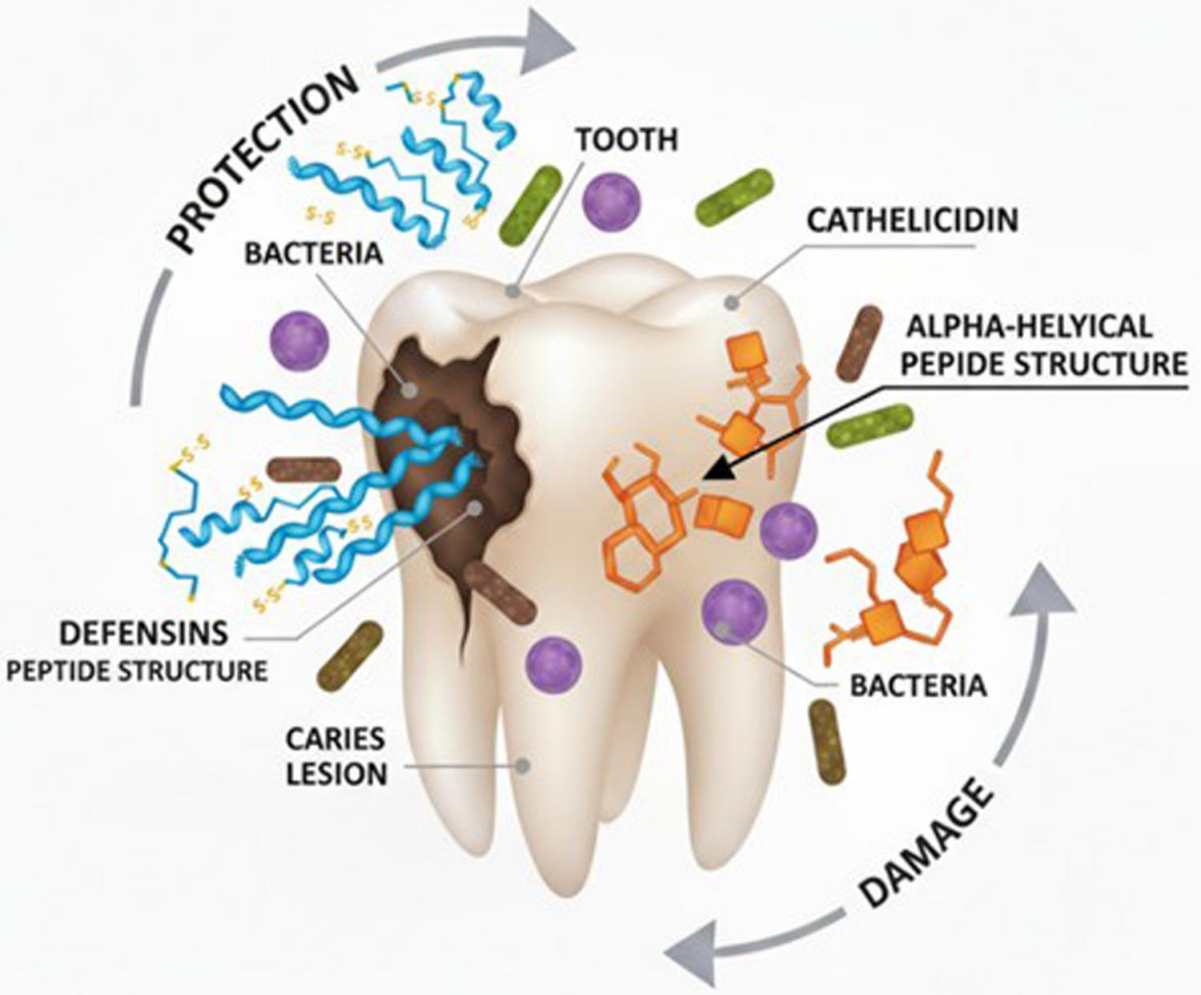
Cathelicidin and defensin protecting the tooth.

Dental caries is a prevalent disease. The Global Oral Health Data Bank states that the prevalence ranges from 49% to 83% across various countries. Regardless of age, it has been shown to harm the quality of life (16). Vitamin D is gaining importance in maintaining dental health; deficiency of vitamin D (VDD) has been proven to affect oral health by the production of salivary AMPs. Studies have shown that VDD is associated with periodontal health, gingival inflammation, and dental caries (17) (Figure 1).

Metabolic syndrome is anticipated to be associated with Vitamin D deficiency. However, research in Indian populations has shown contradictory results. According to the National Cholesterol Education Program Adult Treatment Panel III (NCEP ATP III) definition, the patient with metabolic syndrome should present with three or more of the criteria (15, 18) as mentioned in Table 1. Three criteria have been followed, such as the circumference of the waist, diabetic, and hypertensive patients on drugs. According to our study, the distribution of gender was comparable between the two groups. Males constituted 52.5% of the metabolic group and 45% of the non-metabolic group, while females accounted for 47.5% and 55%, respectively. There was no notable difference in gender distribution between the groups (Table 4).

Vitamin D3 showed no significant differences among the groups, with a *p*-value of 0.577 (Table 1). A relatively higher proportion of deficiency was observed in the metabolic group (27.5%). The study by Wang et al. (19) concluded that vitamin D3 concentration is independent of the elderly metabolic group. Gradillas-García et al. (20) found a significant association between vitamin D deficiency and metabolic syndrome among the adult population from the community of Madrid, which was contradictory to our findings. Vitamin D3 levels also depend upon the skin's exposure to the sun (21).

**Figure 2:**
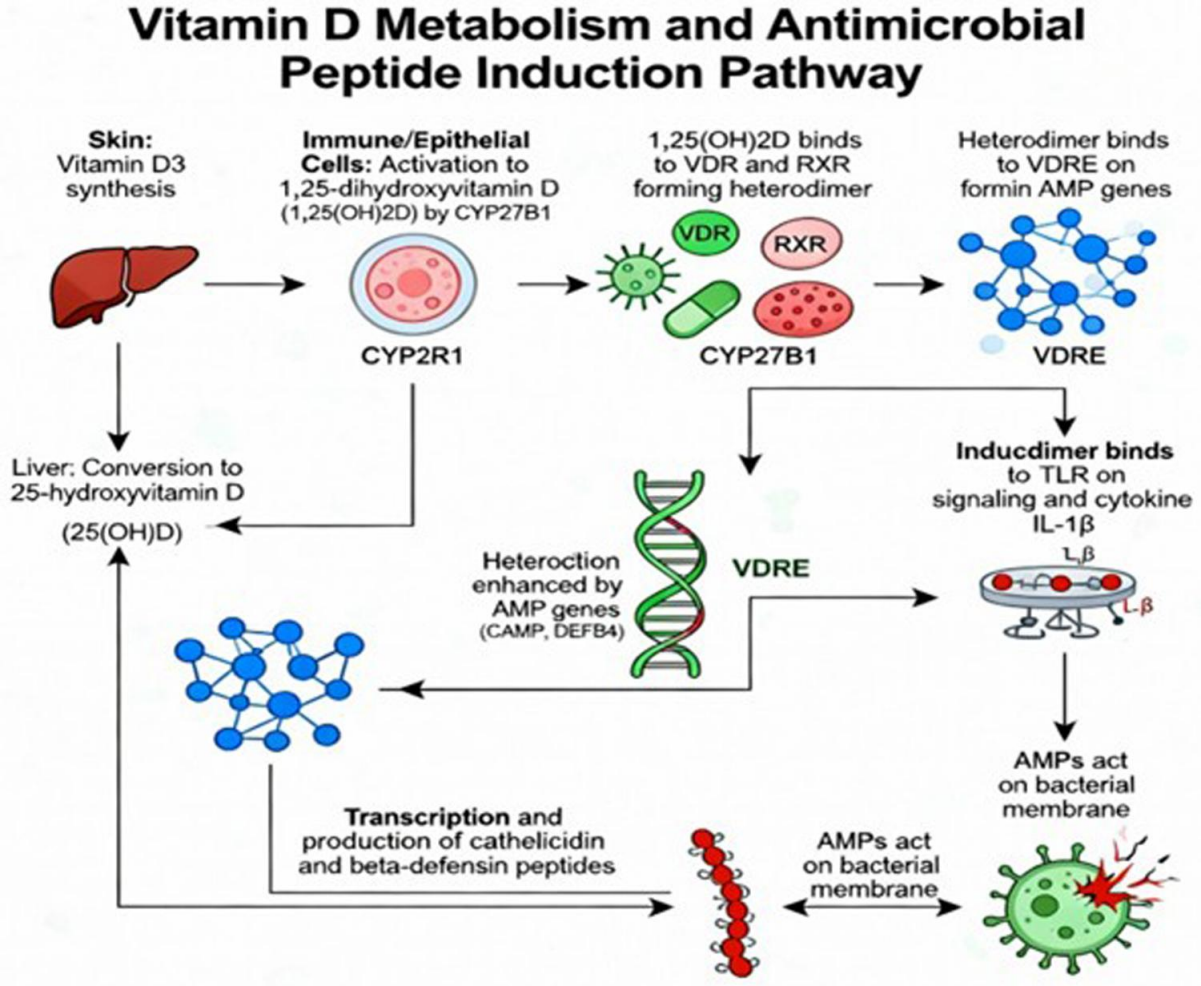
AMPs induction pathway.

The major antimicrobial peptides (AMPs) in the oral cavity are cathelicidin LL37 and β-defensin 2, found and expressed in the soft tissues of the oral cavity. β-Defensin 2 is expressed and derived from oral epithelium, while neutrophils are the main source of cathelicidin (22). AMPs make up the initial line of defense in the oral cavity (23, 24). AMPs exhibit a synergistic interactive activity. The mechanism of action is expressed through killing the cells, by lysis of the cell membrane, and disrupting the cell metabolism (25, 26). Cathelicidin, LL-37, exhibits a broad antimicrobial activity on both cariogenic and periodontopathic organisms (22). AMPs maintain the normal flora in a balanced oral condition (27). Salivary AMPs are likely to serve as proteomic biomarkers for several oral diseases, such as dental caries (28) (Figure 2).

**Figure 3:**
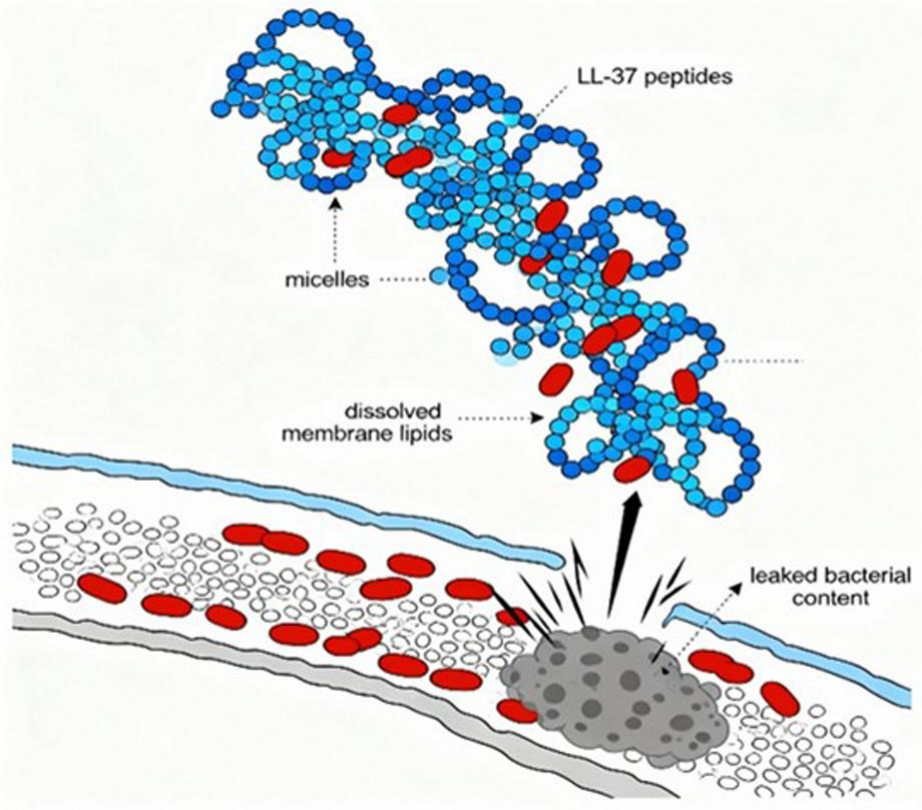
Mechanism of action of cathelicidin—LL37.

Cathelicidins are the first line of peptides that encounter pathogens. The human LL-37 peptide performs multiple functions that help in the maintenance of oral health. The antibacterial property of cathelicidin is observed, as it destabilizes the bacterial cell membrane with proteins. The role of these peptides is not restricted to antibacterial activity, but also it is antiviral and antifungal in actions (29, 30) (Figure 3).

Our study did not show any significant difference between the presence of cathelicidin among metabolic and non-metabolic groups, with a *p*-value of 0.658 (Table 3). Al-Ali et al. (22) found a negative correlation among the children with caries and the non-carious group (31). Almusaileekh et al. established the relationship between cathelicidin and dental caries, where the study showed that cathelicidin was significantly reduced after the extraction of the carious tooth, indicating that it plays an important role in the innate immune system (32). Similarly, Dale et al. conducted a study among school children, which showed increased levels of cathelicidin in the no-caries group than in those with caries. Our study recorded lower DMFT scores among the non-metabolic group, exhibiting no statistical significance among the groups (Table 6). The periodontal pockets and expression of cathelicidin were significantly higher among the metabolic group. Yılmaz et al. (42) concluded in the study that cathelicidin plays an important role in maintaining gingival health (33) which is in line with our study.

**Figure 4:**
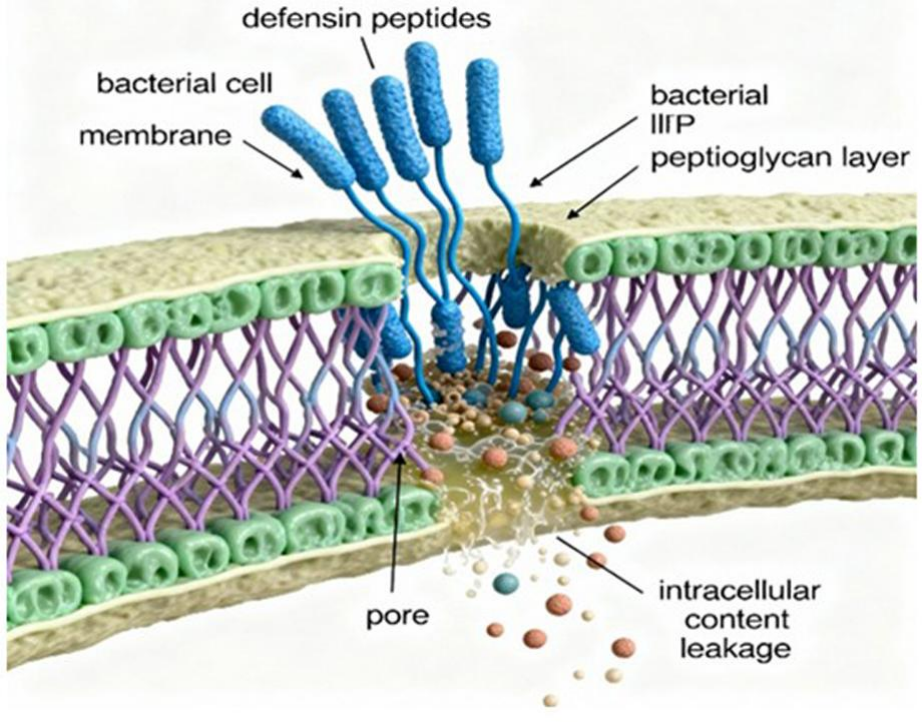
Mechanism of action of defensin 2 on the bacteria.

β-Defensin 2 is a cationic antimicrobial peptide of a large family that is an essential domain of innate immunity. It acts as an important key effector molecule in host defense, exhibiting broad-spectrum antimicrobial activity (34) (Figure 4). β-Defensin 1–3 has a widespread distribution in the human body and has antimicrobial activity against microorganisms. β-Defensin 2 is expressed by epithelial cells in the oral cavity, upon stimulation with pro-inflammatory cytokines, such as IL-1, TNF, and IFN, and microorganisms (35, 36) Our study demonstrated no significant difference among the groups with a *p*-value of 0.179, (Table 4), but the metabolic group expressed a significantly higher β-defensin 2 compared with the non-metabolic group. Jurczak et al. conducted a study evaluating the early childhood caries progression, which showed that β-defensin 2 was higher among the caries group, showing the greatest microbial activity toward Gram-negative bacteria (37). β-Defensin 2 was proven to be expressed during aggressive periodontitis against inflammatory markers, TNF-α (38). In our study, periodontal pockets and expression of TNF-α were significantly higher among the metabolic group, showing higher expression of β-defensin 2 (Table 4), indicating that the metabolic group exhibited poor oral conditions with a high DMFT score (Table 6) and higher periodontal pockets (Table 7).

Tumor necrosis factor alpha TNF-α is a potent inflammatory marker that initiates an immune response. The expression of TNF-α is a process of the host response to pathogenic factors. Our study exhibited the mean salivary TNF-α level of 0.46 ± 0.35 ng/mL in the metabolic group and 0.62 ± 0.42 ng/mL in the non-metabolic group (Table 5). Although TNF-α levels appeared slightly higher in the non-metabolic group, with no statistical significance. A study conducted by Eivazi et al. on salivary TNF-α concentrations among healthy and periodontitis subjects showed a higher level of TNF-α among the healthy patients, which is similar to what our study showed: the higher-level expression of TNF-α among the non-metabolic group. The probable reason could be the greater number of teeth present, which was statistically significant among the missing teeth between the groups, with a *p*-value of <0.0001 (Table 5). Studies have also shown that individuals with metabolic syndrome with chronic low-grade inflammation may lead to immune dysregulation and a reduction in TNF-α production. Prolonged, intense inflammation acts as a stressor that causes immune cells and macrophages to become functionally impaired, leading to altered cytokine production, however. In contrast to our study, Rathinasamy et al. stated that the mean TNF-α level was marginally higher among the patients with chronic periodontitis, but it was not statistically significant.

### Policy implications

The cross-sectional studies show that vitamin D influences innate immune responses, supports oral tissue health, and modulates inflammation and infection risks, underscoring its relevance in disease prevention and oral health maintenance. The future oral health policies with future implications have to be developed for better oral health and the overall well-being of the patients.

- Nutritional guidelines: Addressing vitamin D deficiency, which is globally prevalent, can be an effective public health measure to reduce oral diseases such as caries and periodontitis by enhancing immune defense in the oral cavity (5, 39).
- Preventive strategies: routine screening for vitamin D status and using saliva as a non-invasive tool for immune marker assessment may help target preventive interventions, especially in populations at high risk for oral diseases (40, 41).
- Oral health programs: integrating vitamin D supplementation and monitoring, alongside education about immune functions of antimicrobial peptides and cytokines, can further preventive oral health policies and improve population-level outcomes (5, 40).

### Limitations of the study

With the study conducted, recruiting the metabolic patients toward the lower age limit was challenging, as the number toward the lower limit was minimal. Inclusion of the indices for the periodontal evaluation should have been considered for better understanding. The study should have a greater number of samples to generalize the results.

## Conclusion

This study highlights the critical interplay between vitamin D, salivary antimicrobial peptides, and cytokines in maintaining oral immunity and preventing oral diseases. Vitamin D plays a pivotal role in modulating innate immune responses by upregulating antimicrobial peptides such as LL-37 and regulating cytokine expression, thereby enhancing the oral cavity's defense against pathogens. The findings of our study underscore the potential of vitamin D optimization as a preventive strategy to reduce the burden of oral diseases. Furthermore, understanding these biological mechanisms provides valuable insights for developing evidence-based oral health policies that integrate nutritional and immunological approaches to prevent disease. This translational perspective supports the inclusion of vitamin D status monitoring and targeted interventions in public oral health programs to improve population-level oral health outcomes. Future research with larger cohorts and longitudinal designs is essential to consolidate these findings and inform clinical and public health interventions. Incorporating vitamin D status monitoring and targeted supplementation into oral health programs could significantly enhance population-level outcomes.

## Data Availability

The original contributions presented in the study are included in the article/Supplementary Material; further inquiries can be directed to the corresponding authors.
