# Correction: Exploring the interplay of vitamin D, salivary antimicrobial peptides, and cytokines in oral immunity and disease prevention: an insight for implications in oral health policy

**DOI:** 10.3389/froh.2026.1801959

**Published:** 2026-04-24

**Authors:** Ashwini Tumkur Shivakumar, Sumana Mahadevaiah Neelambike, Supreeta R. Shettar, G. K. Megha, Sowmya Halasabalu Kalgeri, Varsha D. Shiragannavar, Nirmala G. Sannappa Gowda, Dhakshaini MR, Keshava Prasad, Prasanna K. Santhekadur

**Affiliations:** 1 Department of Conservative Dentistry and Endodontics, JSS Dental College and Hospital, JSSAHER, Mysore, India; 2 Department of Microbiology, JSS Medical College and Hospital, JSSAHER, Mysuru, India; 3 Department of Biochemistry, JSS Medical College and Hospital, JSSAHER, Mysuru, India; 4 Department of Biochemistry, MVJ Medical College and Research Hospital, Hoskote, India; 5 Department of Prosthodontics and Crown and Bridge, JSS Dental College and Hospital, JSSAHER, Mysore, India; 6 Department of Conservative Dentistry and Endodontics, RV Dental College and Hospital, Bengaluru, India

**Keywords:** β-defensin 2, cathelicidin, metabolic syndrome, salivary AMPs, vitamin D

In the original article, the author list, corresponding-author designation, and affiliations were published incorrectly.

In the published article, the author name was incorrectly written as Varsha D Shiragannavar. The correct author name is Varsha D. Shiragannavar.

The author list and the affiliation numbering were incorrectly published as:

Ashwini Tumkur Shivakumar^1,2*,^ Sumana Mahadevaiah Neelambike^3,^ Supreeta R Shettar^3,^ G. K. Megha^3,^ Varsha D Shiragannavar^3,^ Nirmala G Sannappa Gowda^1,^ Dhakshaini MR^4,^ Keshava Prasad^5,^ Prasanna K. Santhekadur^3^*

^1^Department of Conservative Dentistry and Endodontics, JSS Dental College and Hospital, Mysore, India

^2^Department of Microbiology, JSS Academy of Higher Education and Research, Mysuru, India

^3^Department of Microbiology, JSS Medical College, Mysuru, India

^4^Department of Prosthodontics and Crown and Bridge, JSS Dental College and Hospital, JSSAHER, Mysore, India

^5^Department of Microbiology, RV Dental College and Hospital, Bengaluru, India

The correct author list and affiliation numbering are:

Ashwini Tumkur Shivakumar^1*,^ Sumana Mahadevaiah Neelambike^2^, Supreeta R Shettar^2^, G. K. Megha^2^, Sowmya Halasabalu Kalgeri^1^, Varsha D. Shiragannavar^3^, Nirmala G Sannappa Gowda^4^, Dhakshaini MR^5^, Keshava Prasad^6^, and Prasanna K. Santhekadur^3^*

^1^Department of Conservative Dentistry and Endodontics, JSS Dental College and Hospital, JSSAHER, Mysore, India

^2^Department of Microbiology, JSS Medical College and Hospital, JSSAHER, Mysuru, India

^3^ Department of Biochemistry, JSS Medical College and Hospital, JSSAHER, Mysuru, India

^4^ Department of Biochemistry, MVJ Medical College and Research Hospital, Hoskote, Bangalore

^5^ Department of Prosthodontics and Crown and Bridge, JSS Dental College and Hospital, JSSAHER, Mysore, India

^6^ Department of Conservative Dentistry and Endodontics, RV Dental College and Hospital, Bengaluru, India.

The original version of this article has been updated.

